# Surgical Duct-to-Duct Reconstruction: an Alternative Approach to Late Biliary Anastomotic Stricture After Deceased Donor Liver Transplantation

**DOI:** 10.1007/s11605-020-04735-y

**Published:** 2020-07-29

**Authors:** Jens Mittler, Kenneth D. Chavin, Stefan Heinrich, Roman Kloeckner, Tim Zimmermann, Hauke Lang

**Affiliations:** 1 Department of General, Visceral, and Transplant Surgery, University Medical Center Mainz, Langenbeckstrasse 1, 55131 Mainz, Germany; 2 University Hospitals Cleveland Medical Center, 11100 Euclid Avenue, Cleveland, OH 44106-5047 USA; 3 Department of Diagnostic and Interventional Radiology, University Medical Center Mainz, Langenbeckstrasse 1, 55131 Mainz, Germany; 4 First Medical Department, University Medical Center Mainz, Langenbeckstrasse 1, 55131 Mainz, Germany

**Keywords:** Biliary complication, Biliary reconstruction, Bilio-enteric diversion, Hepatico-jejunostomy, Surgical bile duct repair

## Abstract

**Background:**

Bilio-enteric diversion is the current surgical standard in patients after deceased donor liver transplantation (DDLT) with a biliary anastomotic stricture failing interventional treatment and requiring surgical repair. In contrast to this routine, the aim of this study was to show the feasibility and safety of a duct-to-duct biliary reconstruction.

**Patients:**

Between 2012 and 2019, we performed a total of 308 DDLT in 292 adult patients. The overall biliary complication rate was 20.5%. Patients with non-anastomotic or combined strictures were excluded from this analysis. Out of 273 patients after a primary duct-to-duct reconstruction, 20 (7.3%) developed late isolated AS. Seven of these patients failed interventional biliary treatment and required a surgical repair.

**Results:**

Duct-to-duct reconstruction was feasible and successful in all patients. Liver function tests fully normalized and no patient required any form of biliary intervention after surgery. One patient with intraoperative cholangiosepsis was ICU bound for 5 days, and another patient with a subhepatic abscess required percutaneous drainage. There was no perioperative death. The median length of hospital stay was 8 (5–17) days. The median time of follow-up after relaparotomy was 1593 (434–2495) days.

**Conclusion:**

Duct-to-duct reconstruction is a feasible and safe option in selected patients requiring surgical repair for isolated AS after DDLT. This approach preserves the biliary anatomy and avoids the potential side effects of a bilio-enteric diversion.

## Introduction

Biliary reconstruction is known to be the “Achilles heel” in liver transplantation. Biliary complications still contribute substantially to the morbidity and mortality of the transplant procedure and present as either leaks or strictures.^1^ According to their localization, biliary strictures are usually divided into anastomotic (AS), non-anastomotic (NAS), or combined strictures. According to the time of occurrence, AS can be divided into early AS (< 30 days) which is mostly caused by technical failure and late AS (> 30 days) which usually develops after months (or years) and is supposedly due to ischemia of the distal donor bile duct, a prior leak of the anastomosis with subsequent scaring or local inflammation.^2^

In managing biliary AS after DDLT, most centers use a stepwise approach.^3^,^4^ In patients with a primary duct-to-duct reconstruction (choledocho-choledochostomy, hepatico-choledochostomy, or hepatico-hepaticostomy), the first-line treatment of AS is ERCP and repeated balloon dilatation with or without placement of one or multiple stents. Alternatively, a percutaneous approach can be chosen. In patients failing interventional therapy, surgical revision of the biliary anastomosis is indicated. According to the literature, a bilio-enteric diversion in the form of a hepatico-jejunostomy (H-J) is considered the standard of care in this situation.^5^,^6^ Although bilio-enteric diversion is a well-established surgical technique that can provide good long-term outcome in this setting, it is potentially associated with ascending cholangitic infections and loop syndromes which can harm the liver allograft and patient. In contrast, if feasible and safe, a bile duct repair in a duct-to-duct fashion could preserve the biliary anatomy and physiology and avoid the potential side effects of bilio-enteric diversion.

The aim of this study was to demonstrate the feasibility and safety of a surgical duct-to-duct repair instead of a bilio-enteric diversion in patients after DDLT with isolated biliary anastomotic stricture failing interventional therapy and requiring surgical repair.

## Patients and Methods

### Patients and Primary Transplant

Between October 2012 and June 2019, 308 deceased donor liver transplants (DDLT) in 292 adult recipients were performed at our center. The overall biliary complication rate observed in this period in all 292 recipients was 20.5% (60/292). Eight patients who underwent late retransplantation for chronic allograft dysfunction were excluded from this analysis. Also excluded were eleven patients who received a primary bilio-enteric diversion because of a diseased recipient bile duct (mostly PSC patients) or a too short donor bile duct. In the remaining 273 patients, biliary reconstruction was carried out as a duct-to-duct anastomosis in an end-to-end fashion. A t-tube was inserted at the surgeon’s discretion, e.g., in the setting of a caliber mismatch between donor and recipient bile duct or for postoperative monitoring of bile quality in marginal grafts.

### Diagnosis of AS

Out of 273 patients after a primary duct-to-duct biliary reconstruction, twenty (7.3%) developed late isolated AS. None of these patients were diagnosed with a biliary leak earlier on. Median time from transplant to diagnosis of late AS was 234 (94 to 478) days. Out of twenty patients, seventeen were detected with elevated liver function tests (LFTs) upon routine posttransplant follow-up. The remaining three patients presented with febrile episodes of cholangitis. Diagnosis of late AS was confirmed by ERCP in fifteen and by PTC in five patients. Patency of the hepatic vasculature especially the hepatic artery was documented by ultrasound and CT scan in all patients.

### Management of AS

Out of twenty patients with late isolated AS, fifteen were initially managed endoscopically with repeated balloon dilatation and stent placement, and five patients required percutaneous biliary interventions.

**Fig. 1:**
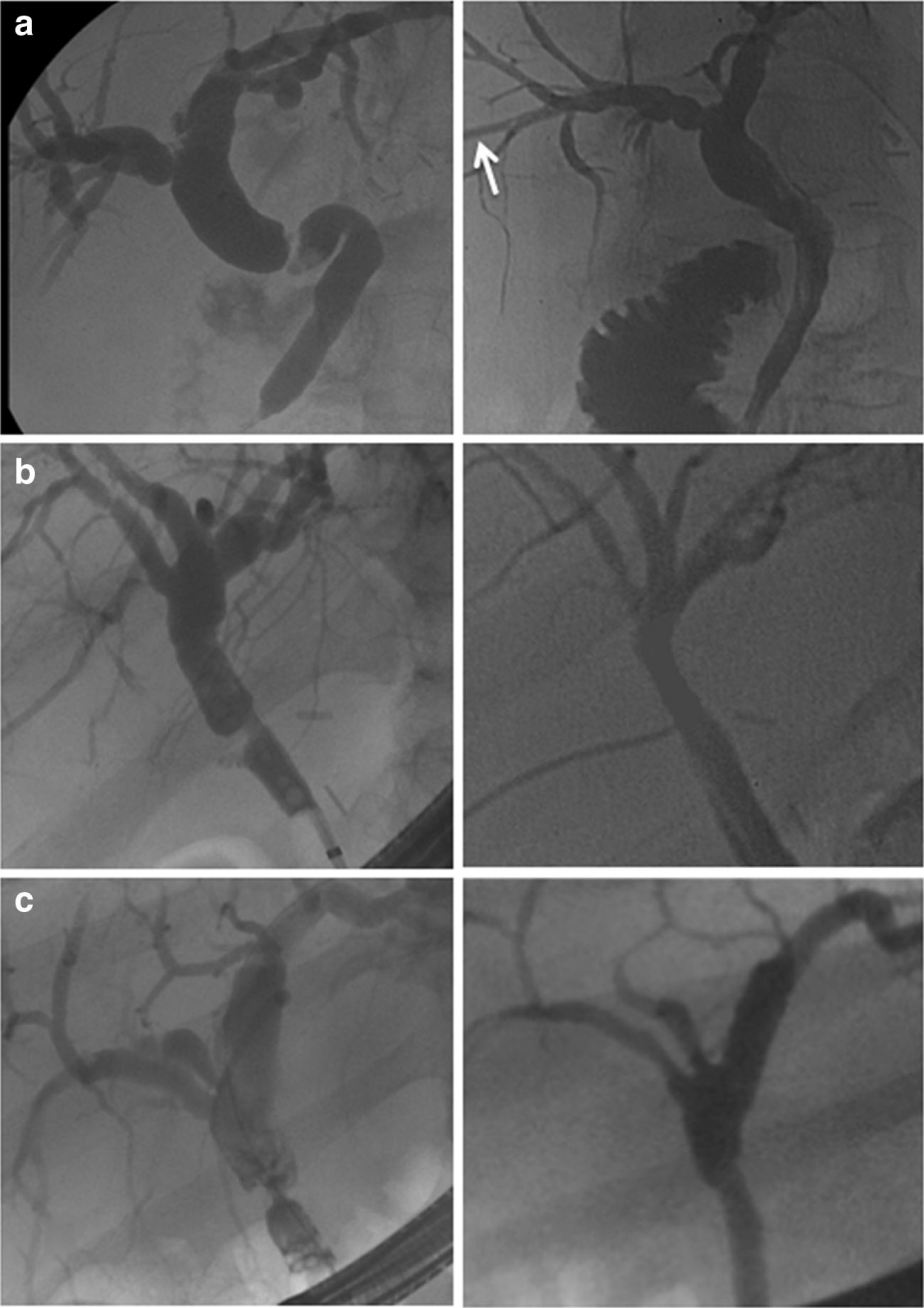
Cholangiograms before (left side) and after (right side) surgical duct-to-duct biliary repair in three patients (**a**–**c**). In patient A, endoscopic stent placement failed due to excessive CBD kinking and preoperative percutaneous intervention became necessary. The PTCD (white arrow) was intraoperatively left in place and removed after 4 weeks. In patient B, the preoperative ERCP and the postoperative t-tube cholangiogram are shown. In patient C, the intraoperatively placed biliary stent was postoperatively removed (pre- and postoperative ERCP)

Seven of twenty patients with late AS failed to respond to interventional therapy after a median of 4 (2 to 8) endoscopic/percutaneous treatment sessions and were scheduled for a surgical revision of the biliary anastomosis. Surgery included a relaparotomy, careful exploration of the liver hilum, and identification of the bile duct. An intraoperative cholangiogram was performed in all cases either through the PTC catheter in place or by introducing a 20G needle into the bile duct. The purpose of the cholangiogram was to show the exact localization and longitudinal extent of the stricture. The stenotic segment was then resected—as much as necessary, as little as possible. Arterial bleeding/oozing from the cutting edge was taken as a surrogate marker of a sufficient blood supply of the donor bile duct and was present in all cases. After careful mobilization of the supraduodenal portion of the recipient bile duct, the biliary reconstruction could be performed using a duct-to-duct anastomosis in an end-to-end fashion in all seven cases (Table 1). In one patient, a t-tube was inserted intraoperatively (Fig. 1b). In another patient, a soft-flex™ biliary stent was placed (Fig. 1c), and in a third patient, the preoperatively inserted PTC catheter was left in place (Fig. 1a). T-tube, stent, and PTCD were each removed about 4 weeks postoperatively. Immunosuppression was continued throughout the perioperative course. One patient on dual immunosuppression with everolimus/low-dose tacrolimus was perioperatively converted to tacrolimus monotherapy.

**Table 1:** Surgical duct-to-duct reconstruction in seven patients with late isolated AS after DDLT

*n*	7
Time from LT to diagnosis of late AS [median (range), days]	234 (94–478)
Initial interventional treatment [*n*]	
Endoscopy alone (ERCP + stent)	5
Percutaneous (PTCD)	2
Median number of interventional sessions	4 (2–8)
Length of bile duct segment resected [median (range), mm]	17 (12–20)
Length of hospital stay [median (range), days]	8 (5–17)
Laboratory recovery (in 7 patients with late AS)	(preop, > 30 days postop)
Total bilirubin [median, mg/dl]	4.8, > 0.9
Alkaline phosphatase [median, U/L]	312, > 146
Gamma glutamyl transferase [median, U/L]	586, > 83
Alanine-aminotransferase [median, U/L]	112, > 34
Aspartate-aminotransferase [median, U/L]	98, > 29
Follow-up after relaparotomy [median (range), days]	1593 (434–2495)

Patient data were prospectively collected in an SPSS™ database and processed using Excel™.

## Results

Seven out of twenty patients with late AS failed interventional treatment and underwent relaparotomy, resection of the stenotic segment, and a duct-to-duct reconstruction as mentioned above. No patient undergoing surgical revision for late AS after DDLT in our center in this time period required a bilio-enteric diversion. The surgical approach was successful in all patients. There were no perioperative deaths. No vascular injury occurred during hilar dissection. One patient developed intraoperative cholangiosepsis which was successfully managed by antibiotics and postoperative intensive care treatment. One patient developed a subhepatic abscess detected on POD #6 requiring percutaneous drainage and antibiotic treatment. The drainage catheter was removed after 9 days. All other patients had an uneventful postoperative recovery. The median length of hospital stay was 8 (5 to 17) days. One month after surgery, the median total bilirubin decreased from 4.8 to 0.9 mg/dL, the median alkaline phosphatase from 312 to 146 U/L, and the median gamma-glutamyl transferase from 586 to 83 U/L. As a consequence of the improved cholestasis, tacrolimus trough levels dropped postoperatively in 3 patients and dosages needed to be increased. No perioperative episodes of rejection were observed. Median time of follow-up was 1593 (434–2495) days. No patient required any further biliary intervention after surgery.

The median length of the resected bile duct segments was 17 (12 to 20) mm. Pathologic examination of the bile duct specimens revealed chronic sclerosing inflammation in all cases without evidence of malignancy.

## Discussion

According to the literature, 5–13% of patients after DDLT develop a stricture of the biliary anastomosis.^2^–^4^,^7^ In our present series of 273 DDLT with primary duct-to-duct reconstruction, the rate of isolated AS was 7.3%. In all cases, AS occurred more than 3 months after the transplant (late AS).

The assumed underlying pathomechanisms of AS and risk factors have been analyzed and discussed in detail elsewhere.^1^,^3^ In patients with late AS, we use a stepwise therapeutical approach as most centers worldwide.^4^,^8^ Overall, 60–90% of AS can be managed interventionally,^3^,^9^ and surgery is indicated only in the setting of interventional treatment failure. As the majority of centers, we consider this approach appropriate as it could be shown that failure of first-line interventional therapy did not affect the outcome of second-line surgery.^10^ The number of interventional sessions is controversial; in our series, patients were scheduled for surgery after 2 to 8 interventional sessions. Concomitant anastomotic problems such as kinking of the bile duct (Fig. 1a), large stones, or biliary casts were reasons to consider earlier reoperation.

The surgical standard of care used in this situation is a bilio-enteric diversion in the form of a Roux-en-Y hepatico-jejunostomy.^10^,^11^ Roux-en-Y hepatico-jejunostomy as described by Hepp and Couinaud in 1956 is a safe, versatile, and well-established surgical procedure to treat a wide variety of biliary problems not only in the liver transplant setting.^12^,^13^ However, it can be associated with jejuno-biliary reflux and the risk of recurrent or chronic ascending cholangitis^14^ which could potentially harm the liver allograft. In contrast, duct-to-duct reconstruction preserves the normal biliary anatomy and physiology of bile flow and has been shown to be feasible and safe in other HPB surgery settings.^15^

In the setting of isolated anastomotic stricture after deceased donor liver transplant, we considered resection of the stenotic segment followed by a redo duct-to-duct reconstruction as an alternative approach to bilio-enteric diversion for the following reasons.

Firstly, the sandglass-shaped strictures were of short longitudinal extent in all cases (see preoperative images in Fig. 1). As shown in Table 1, we never had to resect more than 20 mm of longitudinal bile duct length. An intraoperative cholangiogram was obtained in all cases to exactly localize the stricture and to help determining the extent of resection as much as necessary, as little as possible.

Secondly, there often is some redundant length of the CBD after primary duct-to-duct reconstruction although we try to cut back the donor bile duct during the transplant procedure as short as possible to prevent distal ischemia. Due to this redundancy in bile duct length on the one hand and the limited extent of the stricture on the other, a tension-free approximation of the proximal and distal bile duct margins was possible and considered safe in all cases.

Thirdly, the risk of recurrent stricture after a redo duct-to-duct reconstruction was assumed to be low due to the marked preoperative dilatation of the non-strictured CBD yielding a wide end-to-end anastomosis.

Fourthly, the mobilization of a Roux-en-Y limb can be cumbersome in liver transplant recipients with extensive abdominal adhesions due to pretransplant episodes of spontaneous bacterial peritonitis or previous intestinal surgery (which was the case in our first patient). In contrast, the surgical dissection required for a redo duct-to-duct reconstruction could be limited to the right upper quadrant.

The careful intraoperative evaluation of these four aspects in each individual case led us to perform a duct-to-duct reconstruction rather than a bilio-enteric diversion. In any doubt regarding these criteria, of course, we would not have hesitated to perform a bilio-enteric anastomosis as we do in other clinical settings as an early biliary leak due to a necrotic donor bile duct. In this latter setting, often a much more extended bile duct resection up to the bifurcation or even beyond is needed (precluding an end-to-end reconstruction) as opposed to the short-segment resection required in the setting of isolated AS. It can be argued that also after a duct-to-duct reconstruction, there can be some degree of duodeno-biliary reflux in these patients who all had multiple previous endoscopic interventions and papillotomy. But in our experience of seven cases with a long postoperative median follow-up of 1593 (434–2495) days, all patients showed a complete restitution of their cholestasis by laboratory criteria, and no patient required any further biliary intervention after surgery.

Our results, even though in a small number of patients, demonstrate the feasibility and safety of a duct-to-duct reconstruction in selected patients after DDLT with an isolated biliary anastomotic stricture failing interventional treatment and requiring surgical repair.

## Abbreviations

AS: Anastomotic stricture
CBD: Common bile duct
CIT: Cold ischemic time
DDLT: Deceased donor liver transplant
ERCP: Endoscopic retrograde cholangio-pancreaticography
LFT: Liver function test
MELD: Model for end-stage liver disease
NAS: Non-anastomotic stricture
POD: Postoperative day
PSC: Primary sclerosing cholangitis
PTC: Percutaneous transhepatic cholangiography
WIT: Warm ischemic time
